# High glucose induces Smad activation via the transcriptional coregulator p300 and contributes to cardiac fibrosis and hypertrophy

**DOI:** 10.1186/1475-2840-13-89

**Published:** 2014-05-05

**Authors:** Antoinette Bugyei-Twum, Andrew Advani, Suzanne L Advani, Yuan Zhang, Kerri Thai, Darren J Kelly, Kim A Connelly

**Affiliations:** 1 Keenan Research Centre for Biomedical Science, St. Michael’s Hospital, 209 Victoria Street, Toronto, ON M5B 1T8, Canada; 2 Institute of Medical Science, University of Toronto, 1 King’s College Circle, Toronto, ON M5S 1A8, Canada; 3 Department of Medicine, University of Melbourne St. Vincent’s Hospital, Melbourne, Victoria, Australia; 4 Division of Cardiology, St. Michael’s Hospital, 30 Bond St, Rm 7-052, Toronto, ON M5B 1W8, Canada

## Abstract

**Background:**

Despite advances in the treatment of heart failure, mortality remains high, particularly in individuals with diabetes. Activated transforming growth factor beta (TGF-β) contributes to the pathogenesis of the fibrotic interstitium observed in diabetic cardiomyopathy. We hypothesized that high glucose enhances the activity of the transcriptional co-activator p300, leading to the activation of TGF-β via acetylation of Smad2; and that by inhibiting p300, TGF-β activity will be reduced and heart failure prevented in a clinically relevant animal model of diabetic cardiomyopathy.

**Methods:**

p300 activity was assessed in H9c2 cardiomyoblasts under normal glucose (5.6 mmol/L—NG) and high glucose (25 mmol/L—HG) conditions. ^3^H-proline incorporation in cardiac fibroblasts was also assessed as a marker of collagen synthesis. The role of p300 activity in modifying TGF-β activity was investigated with a known p300 inhibitor, curcumin or p300 siRNA *in vitro*, and the functional effects of p300 inhibition were assessed using curcumin in a hemodynamically validated model of diabetic cardiomyopathy – the diabetic TG m(Ren-2)27 rat.

**Results:**

*In vitro*, H9c2 cells exposed to HG demonstrated increased p300 activity, Smad2 acetylation and increased TGF-β activity as assessed by Smad7 induction (all p < 0.05 c/w NG). Furthermore, HG induced ^3^H-proline incorporation as a marker of collagen synthesis (p < 0.05 c/w NG). p300 inhibition, using either siRNA or curcumin reduced p300 activity, Smad acetylation and TGF-β activity (all p < 0.05 c/w vehicle or scrambled siRNA). Furthermore, curcumin therapy reduced ^3^H-proline incorporation in HG and TGF-β stimulated fibroblasts (p < 0.05 c/w NG). To determine the functional significance of p300 inhibition, diabetic Ren-2 rats were randomized to receive curcumin or vehicle for 6 weeks. Curcumin treatment reduced cardiac hypertrophy, improved diastolic function and reduced extracellular matrix production, without affecting glycemic control, along with a reduction in TGF-β activity as assessed by Smad7 activation (all p < 0.05 c/w vehicle treated diabetic animals).

**Conclusions:**

These findings suggest that high glucose increases the activity of the transcriptional co-regulator p300, which increases TGF-β activity via Smad2 acetylation. Modulation of p300 may be a novel strategy to treat diabetes induced heart failure.

## Background

Diabetes mellitus represents a global epidemic, with the International Diabetes Federation projecting that the prevalence of diabetes will reach 552 million people by 2030 [1,2]. Despite advances in therapy, diabetes is still associated with significant cardiovascular morbidity and mortality [3,4]. Whilst premature coronary artery disease remains the major cause of morbidity in patient with diabetes, an entity known as diabetic cardiomyopathy exists, which is defined as diabetes induced alterations in structure and function in the absence of ischemic heart disease, hypertension or other co-morbidities [5,6]. Increased extracellular matrix production and left ventricular hypertrophy (LVH) are prominent features of diabetic cardiomyopathy regardless of whether cardiac function is preserved or reduced [7-11].

Transforming growth factor β1 (TGF-β1) is a pro-sclerotic cytokine that is consistently implicated in organ fibrosis and hypertrophy [12,13]. TGF-β1 is over-expressed in hypertrophic myocardium during the transition from stable hypertrophy to heart failure [14], and up-regulation of TGF-β1 correlates with the degree of fibrosis in the pressure overloaded heart [14]. We have previously shown, in a clinically relevant animal model of diabetes induced heart failure with preserved ejection fraction, the diabetic m(Ren2)27 rat, that increased interstitial fibrosis and cellular hypertrophy is mediated by increased TGF-β1 activity and Smad2 phosphorylation [15,16]. However, unclear at present are the mechanisms by which high glucose mediates the increased TGF-β1 activity and downstream canonical Smad signaling.

p300, a transcriptional co-regulator with intrinsic lysine acetyltransferase activity, is an essential component for Smad-dependent TGF-β-induced extracellular matrix protein collagen synthesis and profibrotic response [17]. An emerging body of work demonstrates that the acetylation/de-acetylation of proteins rivals phosphorylation/dephosphorylation in importance as a modulator of protein function [18,19]. Indeed, upregulation of p300 acetyltransferase activity has been implicated in the pathogenesis diabetes induced renal dysfunction [20,21], cardiomyocyte hypertrophy and extracellular matrix production [22-25], along with glucose induced changes in gene expression in endothelial cells [26,27].

p300 is also known to acetylate Smad2 in a TGF-β1 dependent fashion. Acetylation of a specific lysine residue (Lys^19^) in the Mad homology 1 (MH1) domain of Smad2 induces a conformational change, thereby facilitating DNA binding and transcription [28,29].

Therapeutic strategies to modify acetylation activity have predominantly focused upon curcumin, a low molecular weight polyphenol compound whose safety, tolerability and lack of toxicity at high dose has been well established in both rodent and human studies (doses up to 12 g/day). Curcumin has been shown to act as an inhibitor of the histone acetylase p300 [2,26,30], but also demonstrates anti-oxidant, anti-inflammatory [1,3] and anti-proliferative activity [4,5,31]. Furthermore, curcumin has been demonstrated to modify other signalling cascades implicated in cardiac hypertrophy such as p-38 /PKC/MAPK [6,9,10,32]. Of relevance to this proposal, inhibition of p300 by curcumin, reduced cardiac hypertrophy and improved cardiac function in post MI and pressure overload models of disease, without evidence of toxicity [19,30]. However, whether high glucose induced changes in TGF-β1 activity are dependent upon p300 mediated Smad2 acetylation, and the effect of p300 inhibition upon Smad acetylation in a clinical relevant model of diabetic cardiomyopathy is unknown.

Accordingly, we hypothesized that high glucose enhances activity of the transcriptional co-activator p300, leading to activation of TGF-β via acetylation of Smad2, and that by inhibiting p300, TGF-β activity will be reduced and heart failure prevented in a clinically relevant animal model of diabetic cardiomyopathy. The role of p300 activity in modifying TGF-β activity was investigated with a known p300 inhibitor, curcumin or p300 siRNA *in vitro*, and the functional effects of p300 inhibition were assessed *in vivo* using curcumin.

## Methods

### Cell culture studies

To determine the role of high glucose (HG, 25 mmol/L) in regulating cardiac myocyte p300 activity, rat H9c2 transformed cardiomyoblasts obtained from American Type Culture Collection were exposed to normal glucose (5.6 mmol/L) or high glucose (25 mmol/L) for 48 hrs. Lysine acetyltransferase (KAT-p300) activity was measured as per the manufacturer’s instructions (Catalog No 56100, Active Motif, Carlsbad, CA). The experiment was then repeated with pretreatment of cells using a p300 inhibitor, curcumin 25 μM [22] or p300 siRNA. For siRNA studies cells were transfected (Lipofectamine 2000, Invitrogen, Carlsbad, CA) with 100 nM small interfering RNA (siRNA) for selective silencing of p300 [22]. After 48 hrs, the transfection solution was discarded and cells washed. Following this, p300 activity was assessed described above. Scrambled siRNA and mannitol were used as control for all experimental conditions.

### ^3^H-proline incorporation assay

Neonatal cardiac fibroblasts were isolated from the hearts of 1 day old Sprague–Dawley rat pups as previously described [16]. For stimulation experiments, 5 ng TGF-β1 and/or high glucose (25 mmol/L) with or without curcumin at 25 μM was used. H9c2 cells were then incubated with ^3^H-proline (1 mCi/well, L-[2,3,4,5-^3^H]-proline; Amersham Biosciences) for 48 hrs. Incorporation of exogenous ^3^H-proline was measured using a liquid scintillation counter (LS 6000 Beckman Coulter Canada Inc., Mississauga, ON, Canada) as previously described [33].

### Smad acetylation

H9c2 cells from ATCC were pre-incubated with 25 μM curcumin for 4 hrs prior to being stimulated with 5 ng TGF-β1 for 24 hrs. Total protein was extracted from cells using a lysis buffer. Smad2 was immune-precipitated using a goat polyclonal anti-Smad2/3 antibody. Acetylation of Smad2 was assessed by Western blotting using a rabbit anti-acetyl-lysine antibody (Cell Signaling Technology, Danvers MA).

### Western blotting

Total protein was extracted with ice-cold radioimmunoprecipitation buffer (Santa Cruz) containing a protease inhibitor mixture and quantified with a Bio-Rad Protein Assay Reagent. Protein samples were then separated by SDS-PAGE and transferred onto nitrocellulose membranes (Invitrogen). Membranes were blocked with 5% skim milk in TBS-T and probed with p300/GAPDH antibodies (Santa Cruz) or antibodies from Cell Signaling Technology (Phospho-Smad2, 3101; Smad2/3, 3102; Acetylated Lysine, 9441; Acetyl-histone H3 K9/K14, 9677). Goat anti-rabbit secondary antibody conjugated to horseradish peroxidise (Bio-Rad) was subsequently used and signal was visualized with an enhanced chemiluminescence western blotting detection kit (GE Healthcare).

#### In vivo study

To explore the effects of p300 inhibition in diabetes induced HFPeF, we used the homozygous TGR(mRen-2)27 rat, a transgenic rodent model that develops cardiac dysfunction following the induction of streptozotocin-diabetes [16,34]. At six weeks of age, male TGR(mRen-2)27 rats (n = 8 per group) were randomized to receive either 55 mg/kg of streptozotocin (STZ; Sigma, St Louis, MO, USA) diluted in 0.1 M citrate buffer pH 4.5 or citrate buffer alone (non-diabetic) by a single tail vein injection following an overnight fast. Once diabetes was confirmed, animals were randomized to receive no treatment or curcumin 2%, admixed in chow. Diabetic animals received 2–4 units of isophane insulin (Humulin NPH, Eli Lilly, NSW, Australia) 3 times per week to promote weight gain and to reduce mortality. Each week, rats were weighed and blood glucose was determined by glucometer (AMES, Bayer Diagnostics, Melbourne, Australia).

Animals were housed at constant room temperature (21 ± 1°C) with a 12 hour light/dark cycle and were fed standard rat chow and water *ad libitum*. At 6 weeks post randomization, animals were anesthetized (1.5% isoflurane admixed in 100% 0_2_). The abdomen, neck and chest were shaved, followed by *in-vivo* left ventricular pressure-volume (PV) loop acquisition. Animals were then euthanized, and their heart and lungs excised. All animal studies were approved by the hospital’s animal ethics committee at St Michael’s Hospital, Toronto, Ontario Canada in accordance with the Guide for the Care and Use of Laboratory Animals (NIH Publication No. 85–23, revised 1996).

### Cardiac catheterization

Cardiac catheterization was performed, as previously published [35]. Using the pressure conductance data, a range of functional parameters were then calculated (Millar analysis software PVAN 3.4). These included: end diastolic pressure (EDP), end systolic pressure (ESP), the slope of the end diastolic pressure volume relationship (EDPVR), the slope of the preload recruitable stroke work relationship (PRSW) [36], defined as the relationship between stroke work (SW) and end diastolic volume (EDV), where stroke work is the pressure-volume loop area for each beat.

### Histopathology

Paraffin embedded sections, each 4 μm thick were examined in at least 6 animals per group. The accumulation of matrix was quantified on picrosirius red stained heart sections using computer-assisted image analysis in a blinded fashion, as previously reported [37]. The extent of cardiac myocyte hypertrophy was determined on haematoxylin and eosin stained sections, as adapted from the methods described by Kai and colleagues [38], and reported as previously published [16].

### Quantitative real time RT-PCR

Smad7 gene expression was measured using whole hearts and quantified using ABI Prism 7000 Sequence Detection System (Applied Biosystems, Foster City, CA) according to the manufacturer’s instructions, as previously described [39]. Primer and probe sequences were:

Smad7 forward AGGCTCTACTGTGTCCAAGA; reverse GACTTGATGAAGATGGGGTA. RPL32 forward CAA CAT TGG TTA TGG AAG CAA CA; reverse TGA CGT TGT GGA CCA GGA ACT. The fetal gene program was assessed by RT qPCR as previously reported [15].

Data output was analyzed using the Applied Biosystems Comparative CT method (Applied Biosystems User Bulletin #2). Experiments were performed in triplicate for each sample and no template controls were added to ensure that amplification was not due to contamination of other components within the PCR mixture.

### Statistical analysis

Results are expressed as mean ± SEM. A D’Agostino & Pearson omnibus test was used to determine data distribution. Differences between groups were determined by ANOVA with Neuman Kuels post-hoc comparison, unpaired t-test, Kruskall Wallis test or 2 way ANOVA where appropriate. A value of *p <* 0.05 was considered statistically significant. Statistical analysis was performed using GraphPad Prism 5 (GraphPad Software Inc., San Diego, CA).

## Results

### High glucose increases p300 activity

**Figure 1:**
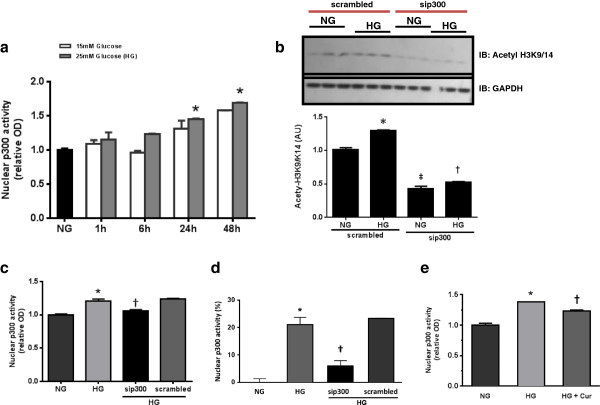
**HG induces nuclear p300 activity and H3K9 acetylation.** p300 activity and H3K9 acetylation are reduced by siRNA directed at nuclear p300 in H9c2 cardiomyoblasts. **(a)**; Nuclear p300 activity was increased in HG media in a time dependent manner, maximal around 48 hours, as is H3K9 acetylation. **(b)**, **(c)** and **(d)**; p300 siRNA reduces HG enhanced nuclear p300 activity to a similar degree to curcumin **(e)**, with an ~18% relative reduction in activity. (*^‡^p < 0.05 c/w NG, ^†^p < 0.05 c/w HG).

To assess whether high glucose alters p300’s acetyltransferase activity *in vitro*, p300 activity was assessed in rat cardiomyoblast (H9c2) cells. Both 15 and 25 mmol/l of glucose was associated with a time dependent increase in p300 activity, maximal at 48 hours (Figure 1a). We also assessed whether high glucose induced H3K9 acetylation, as a marker of p300 mediated histone acetylation [40]. HG significantly increased H3K9 acetylation (Figure 1b).

High glucose not only increased p300 activity, but also increased p300 mRNA and protein levels (Additional file 1: Figure S1). Blockade of p300 gene expression with p300 specific siRNA resulted in a significant decrease of p300 mRNA, protein and activity under high glucose conditions, along with a reduction in H3K9 acetylation (Figure 1b, c and d; Additional file 1: Figure S1). Neither scrambled siRNA nor mannitol, which was used as an osmotic control, altered p300’s acetyltransferase activity. We then assessed whether increased p300 activity secondary to high glucose would be reduced with the polyphenol compound curcumin. Pretreatment of cells with curcumin, followed by high glucose stimulation, resulted in a significant decrease of p300’s acetyltransferase activity (Figure 1e).

### Curcumin inhibits high glucose and TGF-β induced collagen production

**Figure 2:**
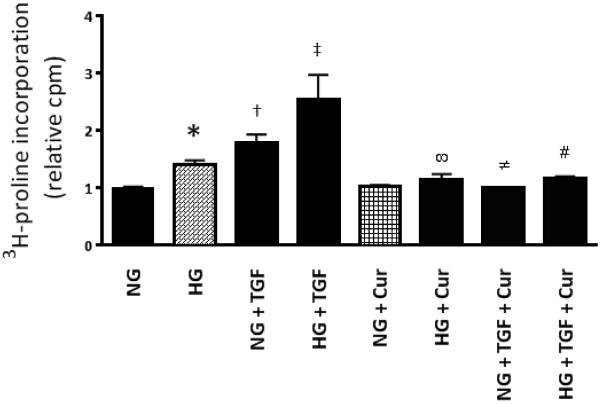
^**3**^**H-proline incorporation as a marker of collagen synthesis: HG and TGF-β increased collagen production, in a synergistic manner in cardiac fibroblasts.** Curcumin at 25 μM reduced collagen synthesis in both HG and TGF-β stimulated H9c2 cells. (*^†^p < 0.01 c/w NG, ^‡^p < 0.01 c/w HG/TGF, ^∞^p < 0.01 c/w HG, ^≠^p < 0.01 c/w NG + TGF, ^#^p < 0.01 c/w HG + TGF)

Given that Smad2-mediated transcription requires p300 lysine acetyltransferase activity [15], we sought to determine the functional effects of p300 inhibition upon high glucose and TGF-β induced collagen synthesis. As expected, neonatal rat fibroblasts exposed to high glucose media or TGF-β showed significantly high levels of ^3^H-proline incorporation, with both high glucose and TGF-β having a synergistic effect (p value for interaction <0.001). However, in the presence of curcumin, ^3^H-proline incorporation induced by high glucose, TGF-β or their combination was significantly reduced (Figure 2).

### Curcumin reduces Smad acetylation

**Figure 3:**
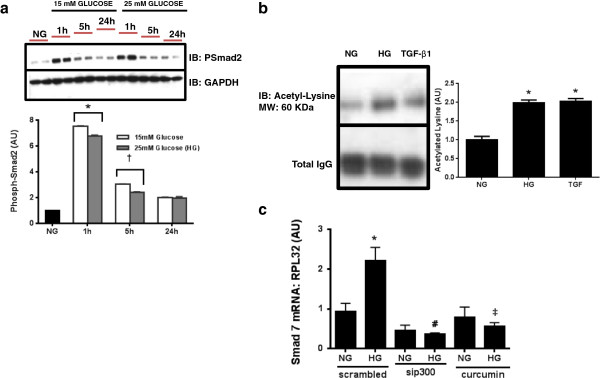
**High glucose increases Smad2 phosphorylation in H9c2 cells.** This occurred in a time dependent manner at two different concentration of glucose **(a)**. HG induced Smad2 acetylation as did TGF-β stimulation **(b)**. Smad7, an inhibitory Smad, increases as TGF-β/Smad activity increases. HG induced TGF-β activity, assessed by Smad7 mRNA. Both p300 siRNA or curcumin reduced Smad7 mRNA, suggesting a reduction in TGF-β activity **(c)**. (*p < 0.05 c/w NG, ^†^p < 0.05 c/w 1 h, ^#‡^p < 0.05 c/w HG scrambled).

**Figure 4:**
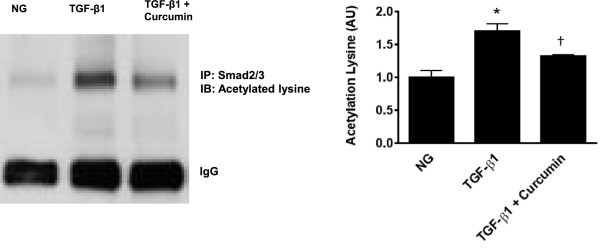
**Curcumin reduces lysine acetylation in TGF-β1 stimulated H9c2 cardiomyoblasts.** Pretreatment with 25 μM curcumin reduced Smad2 acetylation in TGF-β1 stimulated H9c2 cells. (*p < 0.01 c/w NG, ^†^p < 0.01 c/w TGF-β1 treated cells p < 0.05).

We then assessed whether curcumin’s inhibitory effects were mediated by posttranslational changes to the TGF-β intracellular signaling protein Smad2. Thus, we looked at the phosphorylation and acetylation status of Smad2 in cardiomyoblast cells stimulated with HG or TGF-β. HG induced Smad2 phosphorylation in a time dependent fashion, and was maximal at 1 hour. As expected, TGF-β stimulation robustly increased Smad2 acetylation (Figure 3a). Both HG and TGF-β stimulation significantly increased both Smad2 acetylation when compared with NG control (Figure 3b). The inhibitory Smad, Smad7 has been shown to increase as TGF-β activity is increased [21,41]. HG resulted in a significance increase in Smad7 mRNA. Treatment with either p300 siRNA or curcumin reduced Smad7 mRNA demonstrating a reduction in TGF-β activity (Figure 3c). Curcumin had no effect upon Smad2 phosphorylation (Additional file 1: Figure S1). Finally, we assessed the effects of curcumin upon H9c2 cells stimulated with TGF-β1. Curcumin therapy significantly attenuated Smad2 acetylation (Figure 4).

### Curcumin attenuates diabetes induced cardiac dysfunction

Induction of diabetes in the (mRen-2)27 transgenic rat was associated with increased plasma glucose, reduced body weight and pulmonary congestion. Treatment with curcumin did not have a significant effect on plasma glucose nor body weight, but was associated with a reduction in cardiac weight as measured by the heart weight/left ventricular (LV) to body weight ratio or heart weight/LV weight indexed to tibial length (Table 1).

**Table 1:** Animal characteristics of TGR(mRen-2)27 rats

	**Control**	**Diabetes**	**Diabetes + Curcumin**
Plasma glucose (mmol/l)	6.5 ± 0.4	30 ± 0.7^a^	29 ± 0.95^a^
Body weight (g)	307 ± 25	170 ± 12^a^	171 ± 13^a^
Tibial length (mm)	37 ± 0.3	28 ± 0.4^a^	31 ± 0.5^a,b^
Heart weight (g)	1.2 ± 0.06	0.6 ± 0.04^a^	0.51 ± 0.05^a^
Heart weight:body weight (mg/g)	4.2 ± 0.2	3.2 ± 0.12^a^	2.37 ± 0.07^a,b^
HW:TL (g/mm)	3.4 ± 0.16	2.2 ± 0.17^a^	1.76 ± 0.1^a,b^
LV (g)	0.91 ± 0.03	0.52 ± 0.04^a^	0.41 ± 0.03^a,b^
LV:body weight (mg/g)	3.3 ± 0.15	2.8 ± 0.11^a^	2.4 ± 0.07^a,b^
LV:TL (g/mm)	2.47 ± 0.10	1.9 ± 0.1^a^	1.32 ± 0.1^a,b^
Lung weight (g)	1.2 ± 0.7	1.07 ± 0.04^a^	0.84 ± 0.07^a,b^
Lung weight:body weight (mg/g)	0.41 ± 0.01	0.54 ± 0.03^a^	0.51 ± 0.04^a^
LW:TL (g/mm)	3 ± 0.2	3.25 ± 0.06	2.9 ± 0.08

BW is body weight (g); HW is heart weight; TL is tibial length; LW is lung weight.

^a^p < 0.05 compared with control, ^b^p < 0.05 compared with diabetes group.

**Figure 5:**
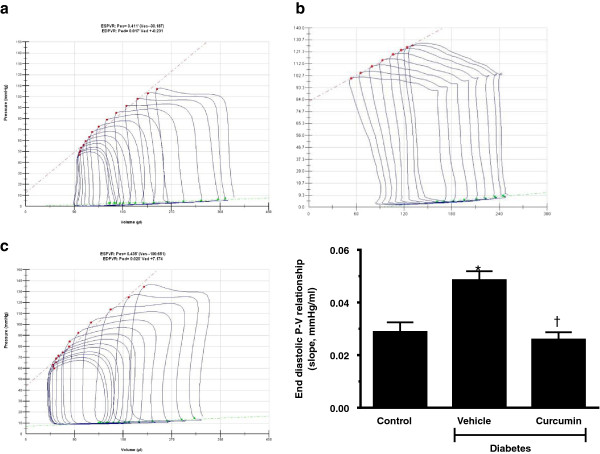
**Curcumin improves chamber compliance in diabetic Ren-2 rats.** Chamber compliance, as measured by the slope of the end diastolic pressure volume relationship (EDPVR, green line bottom right) is significantly increased in diabetic Ren-2 rats **(b)** when compared to control Ren-2 rats **(a)**. Treatment with curcumin significantly improved chamber compliance **(c)**. Quantitative data presented (bottom right). (*p < 0.01 c/w control, ^†^p < 0.05 c/w vehicle treated diabetic Ren-2 rats).

*In vivo* assessment of cardiac function using invasive pressure volume loops demonstrated that diabetes was associated with preserved ejection fraction, but with significant abnormalities in diastolic function. Diabetes was associated with impaired active relation (time constant of relaxation or Tau) along with impaired chamber compliance, as measured by the slope of the end diastolic pressure volume relationship (EDPVR; Table 2). Without effecting heart rate or systolic blood pressure, curcumin treated diabetic rats demonstrated improved early active relaxation (Tau) and chamber compliance (slope of the EDPVR). Systolic function was not altered by curcumin treatment (Figure 5).

**Table 2:** Hemodynamic variables in control and diabetic TGR(mRen-2)27 rats, treated with vehicle or curcumin

	**Control**	**Diabetes**	**Diabetes + Curcumin**
Heart rate (bpm)	348 ± 22	237 ± 14^a^	226 ± 10^a^
ESP (mmHg)	138 ± 15	116 ± 7	114 ± 5.6
EDP (mmHg)	8 ± 1	10 ± 0.5	10 ± 1.1
Tau-Weiss (msec)	18.9 ± 2.4	25.5 ± 0.9^a^	18.5 ± 1.2^b^
ESPVR (mmHg/mL)	0.54 ± 0.23	0.32 ± 0.07^a^	0.46 ± 0.05
EDPVR (mmHg/mL)	0.029 ± 0.004	0.049 ± 0.003^a^	0.026 ± 0.002^b^

ESP is end systolic pressure; EDP is end diastolic pressure; ESPVR is the end systolic pressure volume relationship; EDPVR is the end diastolic pressure volume relationship.

^a^p < 0.05 compared with control, ^b^p < 0.05 compared with diabetes group.

**Figure 6:**
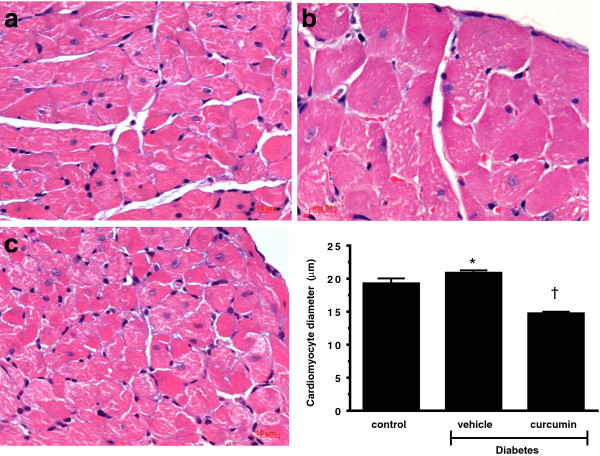
**Haematoxylin and eosin staining of the subendocardial zone to demonstrate cellular hypertrophy.** Diabetic Ren-2 rats **(b)** demonstrated increased cardiomyocyte diameter when compared to control Ren-2 animals **(a)**. Treatment with curcumin reduced pathological hypertrophy **(c)**. Quantitative data presented in bottom right. (*p < 0.01 c/w control, ^†^p < 0.05 c/w vehicle treated diabetic Ren-2 rats).

**Figure 7:**
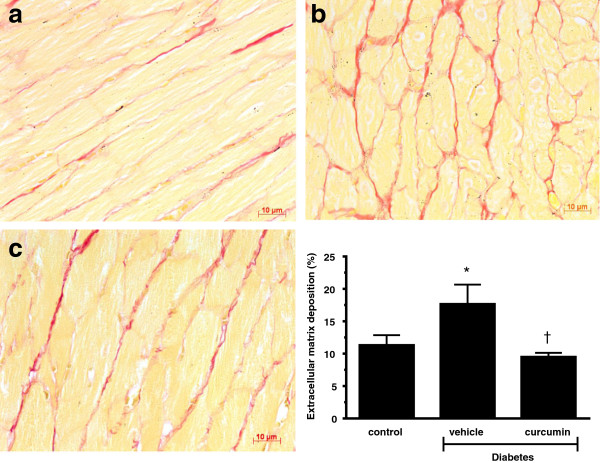
**Picro-red staining of the subendocardial zone to demonstrate fibrosis.** Diabetic Ren-2 rats **(b)** demonstrated increased fibrosis when compared to control Ren-2 animals **(a)**. Treatment with curcumin reduced pathological fibrosis **(c)**. Quantitative data presented in bottom right. (*p < 0.01 c/w control, ^†^p < 0.05 c/w vehicle treated diabetic Ren-2 rats).

As diastolic stiffness is primarily determined by interstitial fibrosis and cellular hypertrophy, we assessed the effect of curcumin upon extracellular matrix content and cell size. Curcumin treated animals demonstrated a reduction in cardiac fibrosis and cellular hypertrophy when compared to diabetic counterparts (p < 0.05; Figures 6 and 7).

In keeping with this finding, activation of the fetal gene program, a marker of cardiomyocyte hypertrophy [22,23,31], was found in the diabetic Ren-2 rats. Atrial natriuretic factor (ANF) and β-myosin heavy chain (β-MHC) were induced, whilst sarcoplasmic reticulum Ca^2+^-ATPase and α-myosin heavy chain (α-MHC) mRNA levels fell in diabetic animals compared to the control counterparts. Treatment with curcumin improved some, but not all aspects of fetal gene program activation. ANF levels were normalized, whilst SERCA 2A levels significantly increased. No change was noted for α-MHC, however β-MHC expression was increased (Table 3).

**Table 3:** Fetal gene program activation as a measure of cardiomycoyte hypertrophy

	**Control**	**Diabetes**	**Diabetes + Curcumin**
ANF	1.02 ± 0.04	1.6 ± 0.28^a^	0.93 ± 0.08^b^
SERCA	1.02 ± 0.10	0.63 ± 0.08^a^	1.72 ± 0.13^b,^^c^
α MHC	1.03 ± 0.07	0.3 ± 0.06^a^	0.2 ± 0.02^a^
β MHC	1.01 ± 0.10	2.2 ± 0.31^a^	5.12 ± 0.36^b,^^c^

ANF is atrial natriuretic factor; SERCA is sarcoplasmic reticulum Ca^2+^-ATPase; α MHC is alpha myosin heavy chain; β MHC is β myosin heavy chain. Units are fold changes compared to control relative to housekeeping gene RPL32a. ^a^p < 0.05 compared with control, ^b^p < 0.05 compared with diabetes group, ^c^p < 0.01 compared with control.

### Curcumin reduces Smad activity in vivo

**Figure 8:**
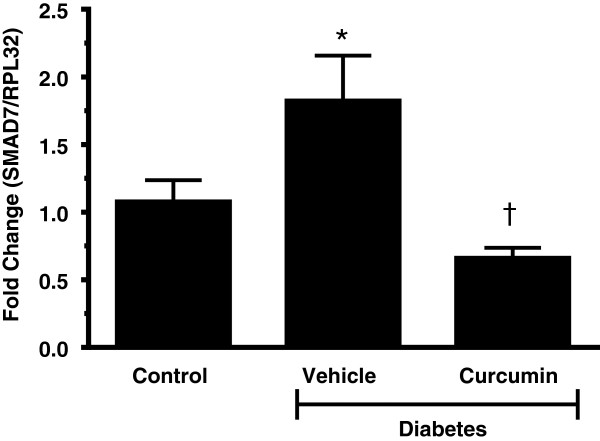
**Diabetes increased Smad activity.** Smad7, an inhibitory Smad, increases as TGF-β/Smad activity increases. Diabetes increased Smad activity compared to control animals. Treatment with curcumin reduced diabetes enhanced TGF-β/Smad activity. Whole hearts were used for analysis. Smad7 mRNA levels expressed as fold change relative to housekeeping gene RPL32. (*p < 0.01 c/w control, ^†^p < 0.05 c/w vehicle treated diabetic Ren-2 rats).

We then performed quantitative real time PCR for Smad7, which is induced when TGF-β activity is increased [26,41]. Diabetic mRen-2 rats demonstrated a 2-fold increase in Smad7 mRNA expression levels that was significantly reduced by curcumin treatment (Figure 8).

## Discussion

In the present study, we demonstrate that high glucose increases the activity of TGF-β via activation of the transcriptional regulator p300. Inhibition of p300 using siRNA or the polyphenol curcumin reduced TGF-β activity, prevented cardiac hypertrophy and reduced fibrosis, independent of glycemic control. These findings suggest therapies aimed at modifying p300 mediated lysine acetylation may be beneficial in treating diabetes related cardiovascular complications.

Despite advances in glycemic control, cardiac risk factor intervention and the management of diabetes induced cardiovascular complications, heart failure with preserved ejection fraction (HFPeF) remains a major cause of morbidity and mortality [26,30,42], with no specific therapeutic interventions [1,43]. Our findings show that cardiac fibrosis and cellular hypertrophy, two cardinal manifestations of diabetes induced cardiac disease, [4,10,31,44] were attenuated by the p300 inhibitor curcumin. Furthermore, by attenuating cardiac fibrosis and hypertrophy, diastolic function was substantially improved in a hemodynamically validated model of diabetes induced HFPeF.

Transforming growth factor beta1 (TGF-β1) is a pro-sclerotic cytokine implicated in organ fibrosis [6,12,13,32,45,46]. Indeed, consistent with our work in diabetes induced cardiac fibrosis [15,16,30,34,47], elevated TGF-β1 expression is consistently found during the transition from stable hypertrophy to heart failure in both experimental models and human heart failure [14,41]. As a result, strategies to reduce TGF-β activity remain an important therapeutic target, however current attempts have been limited by toxicity or off target effects [48-50]. In the present paper, we focused upon inhibition of Smad2, which mediates the intracellular actions of TGF-β receptor activation. Canonical TGF-β1 signaling involves the receptor activated Smad proteins (Smad2 and Smad3), which, upon phosphorylation, associate with Smad4, translocate to the nucleus and act as transcription factors [41,51,52]. However recent data demonstrates that a further level of transcriptional regulation is necessary to mediate TGF-β downstream signaling, involving Smad acetylation [28,29,42]. Indeed, it has come to be appreciated that the post translational modifications of proteins by acetylation and de-acetylation is ubiquitous, comparable to other well described post translational modifications as a key regulator of protein and therefore cell function [19,43]. In the present study we demonstrate that, inhibition of acetylation using siRNA against p300 or the polyphenol curcumin, prevented Smad2 lysine acetylation and inhibited collagen synthesis as evidenced by a reduction in ^3^H-Proline incorporation as a bioassay of fibroblast collagen production.

The lysine acetyltransferase (KAT) p300 is a transcription co-regulator, implicated in the pathogenesis of various disease processes including cardiac hypertrophy and fibrosis [10,22,30,44,53-55]. Importantly, it is directly involved in regulating multiple transcriptional regulators involved in the pathogenesis of diabetes induced cardiomyocyte hypertrophy [12,26,30,45,46]. In a rat cardiomyoblast cell line, we demonstrate that high glucose directly increases p300 mRNA, but more importantly it enhances nuclear p300 activity, and either curcumin or p300 siRNA reduced p300 activity and Smad acetylation. Furthermore, curcumin therapy, reduced Smad activity *in vivo* as measured by a reduction in Smad7 mRNA expression, which is robustly enhanced as a result of increased TGF-β activity [15,16,34,56]. These finding are important as they provide a potential explanation for the enhanced TGF-β activity seen in diabetes [13,47], and demonstrate that the key effects of curcumin are mediated by its ability to inhibit p300 activity. Whilst the exact mechanism behind the enhanced p300 KAT activity found in diabetes is unclear, recent studies focusing upon auto-acetylation [14,57] and the BET bromodomains [48,50,58], suggest that further therapeutic opportunities exist to modify KAT activity in diabetes such as use of the selective bromodomain inhibitors [51,52,58-60].

With the realization that HFPeF carries a similar prognosis to systolic heart failure, but that current therapeutic strategies to improve outcomes have not been successful [28,29,61], understanding the pathophysiology of diastolic dysfunction has become increasingly important [19,62,63]. Diastology encompasses two distinct phases, an early energy dependent phase [22,53,54,64] and a late “passive” filling phase dependent upon the visco-elastic properties of the ventricle [26,30,65]. Diabetes, both in experimental models [16,56] and human studies [10,58] has been shown to impair both active and passive phases of diastole, as measured by the time constant of relaxation (Tau) and the end diastolic pressure volume relationship (EDPVR) respectively. The later passive phase of cardiac filling is primarily determined by myocyte stiffness and fibrosis [5,9,10,58]. In our studies, curcumin, prevented the pathological accumulation of extracellular matrix and reduced cardiac hypertrophy, without effecting blood pressure. These findings manifested as improved chamber compliance and a reduction in the slope of the EDPVR, thus indicating improved diastolic function.

Curcumin, a constituent of the spice turmeric, is a hydrophobic polyphenol with a characteristic yellow color. The safety, tolerability and lack of toxicity at high dose has been well established in rodent and human studies (doses up to 12 g/day) [31,58-60]. Despite clearly acting as an inhibitor of the KAT p300, curcumin has been shown to demonstrate anti-oxidant, anti-inflammatory [1,61], anti-proliferative activity [4,62], anti-hypertrophic and anti-fibrotic activity [30]. As a result, we cannot definitely exclude other potential mechanisms for the effects observed both *in vitro* and *in vivo*[6,64]. Furthermore, we found that curcumin demonstrated a surprisingly narrow therapeutic window (data not shown), with doses exceeding 75 μM *in vitro* resulting in excessive cell death. As a result, the clinical utility of this agent remains doubtful. In order to overcome these limitations, derivatives of curcumin, such as theracurmin have been developed, which demonstrate improved bioavailability and lack of toxicity. These compounds appear promising and are currently in early clinical trials for a variety of indications [7,8,65].

Whilst we focused upon modification of Smad2 as a mediator of downstream TGF-β signaling, TGF-β is one of many proteins involved in modification of the extracellular matrix. Indeed, the interplay between the extracellular matrix, cardiomyocytes, fibroblasts and the key signaling proteins involved remains an area of intense research [13]. Novel matricellular proteins such as thrombospondin-1, and other members of the TGF family such as TGF-β2 play an important role in mediating the fibrotic response in the diabetic myocardium [11,63]. How modification of acetylation may influence these proteins is unclear at the present time. Other therapeutic strategies such as the use of alpha lipoic acid or erythropoietin have been shown to inhibit TGF-β induced extracellular matrix accumulation in diabetic cardiomyopathy [47,66]. These findings suggest that modification of the extracellular matrix, focusing upon TGF-β as a therapeutic strategy in diabetes will likely require multiple complementary strategies in order to counter such well regulated, broad and complex signaling pathways.

Our study has some limitations. Firstly, the transcriptional co-activator p300 modifies a wide variety of cell signaling processes. As a result, whilst we have focused upon one specific target, Smad2 acetylation, we cannot rule out its effects upon multiple other targets. Current therapeutic strategies, such as blockade of the renin-angiotensin system, affect multiple downstream targets [16,18], and microRNAs by definition affect mRNA expression of multiple targets [10,20], thus the lack of specificity does not limit the clinical application of our findings. Secondly, we studied a model of type 1 induced cardiac dysfunction, whereas the majority of patients with diabetes have type 2 diabetes. However, elevated glucose remains the sine qua non of diabetes regardless of type 1 or type 2 forms, and there is no evidence to suggest that p300 activity would be altered differentially in type 1 or type 2 diabetes. Furthermore abnormalities of diastolic function have been documented in both diseases [5,9,10,24,25], thus we believe the findings are relevant. Finally, we did not assess impact of the metabolic abnormalities found in diabetes upon Smad acetylation or p300 function. These will be the focus of further studies and have been the subject of several excellent reviews [27,31].

## Conclusion

In conclusion, these studies demonstrate that high glucose enhances p300 activity and Smad acetylation, thus enhancing TGF-β activity, promoting cardiac fibrosis, hypertrophy and impairing diastolic function. By inhibiting high glucose induced p300 activity, TGF-β activity was attenuated and the key mediators of diastolic dysfunction, fibrosis and cellular hypertrophy were reduced, thus preventing HFPeF. Targeting high glucose induced p300 activity may therefore represent a new therapeutic target in diabetes induced HFPeF.

## Abbreviations

ANF: Atrial natriuretic factor; EDP: End diastolic pressure; EDPVR: End diastolic pressure volume relationship; EDV: End diastolic volume; ESP: End systolic pressure; HFPeF: Heart failure with preserved ejection fraction; HG: High glucose; KAT: Lysine Acetyltransferase; LV: Left ventricular; LVH: Left ventricular hypertrophy; MHC: Myosin heavy chain; MH1: Mad homology 1; NG: Normal glucose; PRSW: Preload recruitable stroke work; PV-loop: Pressure-volume loop; SERCA: Sarcoplasmic reticulum Ca^2+^-ATPase; STZ: Streptozotocin; SW: Stroke work; TGF-β: Transforming growth factor beta.

## Competing interests

The authors declare that they have no competing interests.

## Authors’ contributions

All authors contributed to the conception, design, analysis, and interpretation of data. All authors were involved in drafting/revising the manuscript and in approving the final version of manuscript.

## Supplementary Material

Additional file 1: Figure S1 High glucose increased p300 mRNA and protein levels (a, b); blockade of p300 expression with p300-specific siRNA significantly reduced p300 mRNA and protein levels under high glucose conditions. High glucose induced TGF-β signaling, as assessed by Smad2 phosphorylation levels(c); Curcumin, however, had no effect on Smad2 phosphorylation levels under high glucose conditions. Click here for file
