# Supplementary material for: High glucose induces Smad activation via the transcriptional coregulator p300 and contributes to cardiac fibrosis and hypertrophy
Source: Cardiovasc Diabetol. 2014 May 5;13:89. doi: 10.1186/1475-2840-13-89 (PMC4108062; doi:10.1186/1475-2840-13-89)
Supplement: Additional file 1: Figure S1 — High glucose increased p300 mRNA and protein levels (a, b); blockade of p300 expression with p300-specific siRNA significantly reduced p300 mRNA and protein levels under high glucose conditions. High glucose induced TGF-β signaling, as assessed by Smad2 phosphorylation levels(c); Curcumin, however, had no effect on Smad2 phosphorylation levels under high glucose conditions. [file 1475-2840-13-89-S1.pptx]

## Slide 1
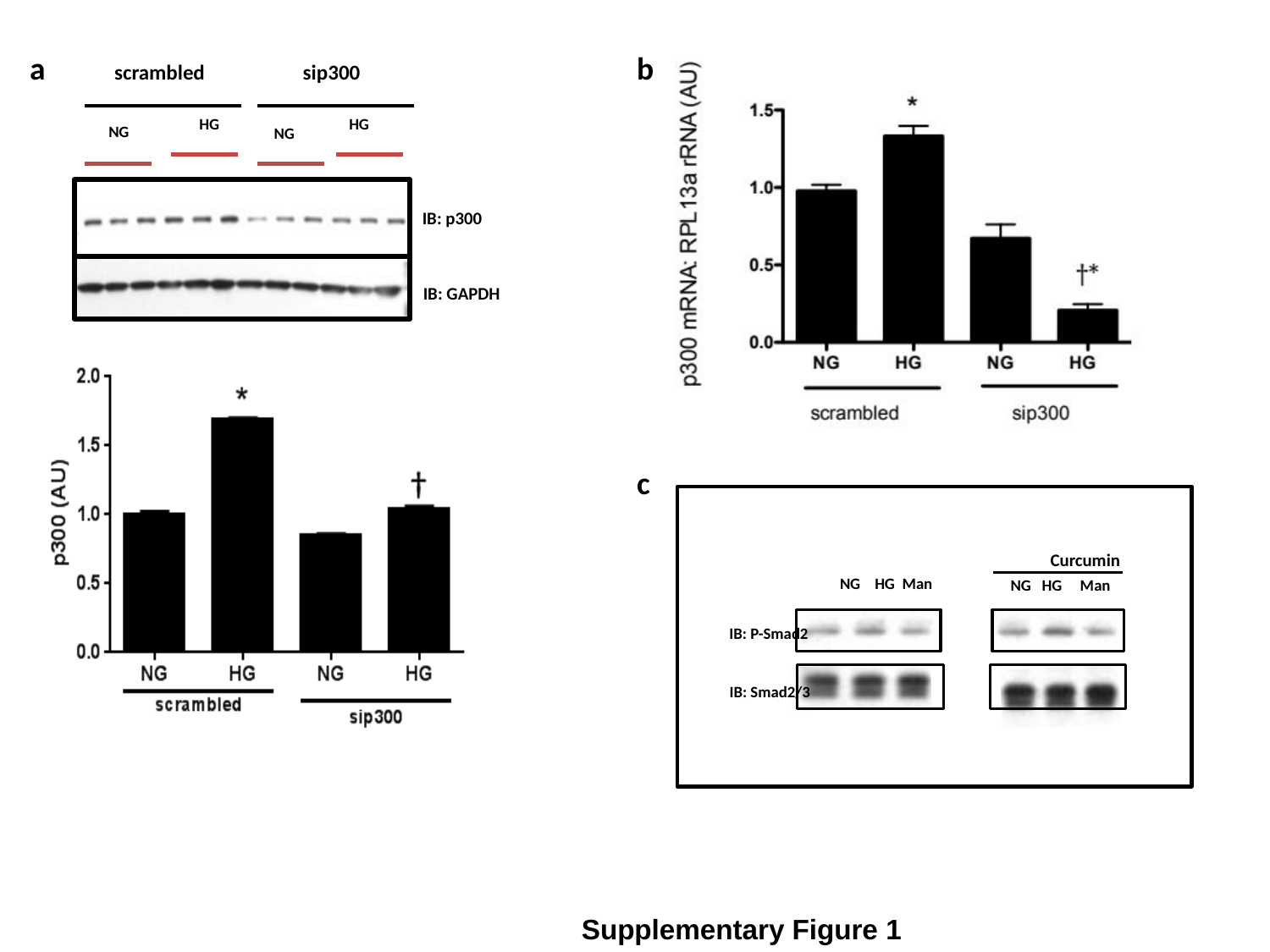

a
b
scrambled
sip300
HG
HG
NG
NG
IB: p300
IB: GAPDH
c
Curcumin
NG HG Man
NG HG Man
IB: P-Smad2
IB: Smad2/3
Supplementary Figure 1
